# Atresia of the Vagina and Uterus

**Published:** 1881-11

**Authors:** A. F. Erich

**Affiliations:** Baltimore, Md.; Professor of Diseases of Women, College of Physicians and Surgeons, Baltimore; Surgeon in Charge of the Maryland Woman’s Hospital, etc.


ATLANTA

### MEDICAL REGISTER.

VOL. I.] NOVEMBER, 1881. [NO. 2

### Original.

### ATRESIA OF THE VAGINA AND UTERUS.—A CLINICAL LECTURE.

BY A. F. ERICH, M.D., BALTIMORE, MD.

Professor of Diseases of Women, College of Physicians and Surgeons, Baltimore; Surgeon in Charge of the Maryland Woman's Hospital, etc.

*Reported for the Atlanta Medical Register.*

GENTLEMEN.—Atresia or complete occlusion may occupy any portion of the genital canal from the *ostium vaginæ* to the internal os of the uterus. It may be congenital or acquired. The most frequent form of congenital atresia is that caused by an imperforate hymen. When there is congenital atresia of the vagina, the defect of development is usually limited to a small section of the vaginal canal. Sometimes there is merely a septum at some portion of the canal between the hymen and the os uteri which prevents the exit of the contained fluid. Cases also occur in which there are several distinct septa, one above the other. The higher up in the vagina the septum is, the greater the danger in operative interference. In some rare cases the only evidence of a vagina discoverable is a fibrous cord extending upward and backward from the pudendal sulcus. In these cases the uterus is also usually undeveloped. The cohesion of the labia sometimes seen in female infants at birth, and more frequently first noticed during childhood, is not due to defective development, but is generally the result of inflammation, either during intra-uterine life or after birth. It is seen most frequently in strumous or illy-nourished children, especially if the laws of cleanliness are neglected. This form of atresia is never complete, a small opening being always found at the anterior commissure, which has evidently been kept patent by the escape of the urine. Through this opening a grooved director or sound can be introduced and the adhesions broken up without much difficulty. A piece of oiled lint should then be placed between the raw surfaces, to prevent their reuniting. Absolute cleanliness, of course, must be insisted upon during the after treatment

In congenital atresia—by which I mean atresia due to arrest of, or faulty development—there are usually no symptoms until the arrival of puberty and the establishment of the functional activity of the ovaries. The appearance of the menses is delayed, but the symptoms indicating their appearance—the so-called menstrual molimina—are more severe than in health. The menstrual blood is poured out, but cannot escape in consequence of the obstruction. The blood accumulating in the uterus or vagina gives rise to the formation of a tumor in the hypogastrium, which, with the absence of menstrual discharge, often raises a suspicion of pregnancy. Uterine colic and tenesmus accompany each menstrual period, becoming more severe as time goes on. These periods of colicky pain recur more and more frequently, until finally there is hardly any interval of rest There is in some cases also painful micturition and defecation In consequence of the pain and loss of rest, emaciation and hectic result, which may terminate in death by exhaustion. The distension of the uterus and vagina may become so great as to eventuate in rupture and consequent peritonitis. Cases of atresia sometimes recur in which the menstrual function, owing to absence or non-development of the ovaries and uterus, never becomes established. In these cases the pain and other subjective symptoms may be entirely absent, and the abnormality only discovered when attempts are made at sexual intercourse.

On physical exploration, the cause of the symptoms is usually easily detected. If the atresia is due to imperforate hymen, a soft, elastic tumor, covered by mucous membrane—the distended hymen—protrudes from the vulva.

Atresia in the vaginal canal may present a similar soft and elastic tumor if the closure simply depends upon a membranous septum. If, however, the occlusion extends some distance the tumor cannot be so distinctly felt from below. With one finger in the rectum and a sound or finger in the bladder, the outlines of the tumor can generally be well made out, and often the extent of the atresia defined.

Acquired atresia is most frequently due to sloughing after prolonged labor or cauterization of the cervix uteri or vagina. It may also follow inflammation and sloughing of the vagina or vulva, which sometimes accompanies diphtheria and other acute infectious diseases in childhood. Lack of cleanliness, especially in strumous or illy-nourished children, may also be a cause. In acquired atresia there are usually scars and contractions in the neighborhood of the occlusion, pointing to precedent inflammation and sloughing.

Considerable difficulty in diagnosis may be caused by a double vagina and uterus, with atresia of one side and perviousness of the other. In such a case there may be regular menstruation through the pervious canal, with accumulation of menstrual fluid on the occluded side. In unilateral atresia the right hand tract is most frequently affected. The diagnosis can in many cases only be verified by the aspirator. If the atresia is in the lower portion of the genital tract the vagina becomes first dilated and its walls hypertrophied. The dilation of the uterus, if it occurs at all, is consecutive. When the accumulation of fluid is large, the Fallopian tubes may also be distended.

The contents of the tumor in atresia usually consist of a dark, syrupy fluid, consisting of blood mixed with mucus, and exfoliated uterine and vaginal epithelium. In children, before the age of puberty has arrived, the accumulation of mucus behind the occlusion may give rise to symptoms demanding an operation for the relief of the imprisoned fluid. This is especially liable to occur in cases of imperforate hymen.

Women who have passed the menopause sometimes suffer from an accumulation of mucus, or muco-purulent fluid, in the uterus, due to atresia of the os following endometritis or the application of caustics to the cervical canal. In other cases the os may be closed by the formation of an epithelial stratum resembling a false membrane, completely occluding the os. This condition is not unfrequently seen in prolapsus of the uterus. A sound can generally be pushed through the obstruction and the passage afterwards dilated.

The indications in all cases of atresia accompanied by symptoms, are to empty the retained fluid, keep the tract pervious, and restore, if possible, the functional capacity of the sexual organs.

When the atresia is due to an imperforate hymen or a thin membranous septum in the vagina, the operation is simple and easily performed. The projecting tumor is punctured with a sharp bistoury, and the opening dilated by the insertion of a finger or a smooth dilator. In dilating the primary puncture, tearing should be preferred to cutting, both to avoid hemorrhage and to prevent rapid healing and reclosure of the opening. When the contained fluid has been evacuated the cavity should be well washed out with carbolized water, and a glass or hard-rubber vaginal plug should be introduced into the vagina. The plug should be removed and cleaned twice a day, and the injections continued till all danger of septic absorption has passed.

To evacuate the retained fluid, in cases in which there is arrest of development or acquired occlusion of the vagina, two methods of operating have been practiced. The method of Amussat consists in opening up the vaginal canal in several operations—by stages, as it were—and to let out the fluid slowly. Dupuytren's operation, modified by Emmett, is, in my opinion, the preferable one, and offers the best chances of relief with least danger to the patient. It consists in making a free opening through the undeveloped or obliterated vaginal tract until the fluid is reached; this is then evacuated, and the vagina—and uterus if involved—syringed out with a warm antiseptic solution. The connective tissue should be torn through with the fingers or a sound and all necessary dissection done with scissors. The reason for using the scissors in preference to the knife is two fold; it causes less hemorrhage and retards the reunion of the cut surfaces and subsequent contraction of the canal. In making the dissection one or two fingers must be kept in the rectum and a properly held sound in the bladder, to guard these organs against injury. As little cutting as possible should be done. It will generally be possible to open a sufficient passage by tearing with the finger nail or sound.

The progress of the case after the operation must be closely watched. The vagina or uterus must be washed out with carbolic acid, or other antiseptic solution, twice or three times a day—oftener if necessary—to prevent absorption of decomposing material and septicæmia. Metritis and pelvic cellulitis are also complications which you must look out for and prepare to treat properly when they arrive. The newly constructed vagina must be kept constantly distended with the vaginal plug, as re-contraction is very liable to occur.

The patient who appears before you to-day, and whose case has given the occasion for the foregoing remarks, applied for admission into the Woman's Hospital three months ago. The history which she has given is entered on the hospital records, of which I will now read you an abstract:

"L. W., colored, 17 years of age, menstruated for the first time when 13 years old. She is unmarried but has had one child, which was born three years ago. If she is correct in her chronology her pregnancy must have begun shortly after the first appearance of menstruation. Since the birth of her child she has had no menstrual discharge. She is of spare physique, but has always lived an active life. There is no history of an exceptionally difficult labor. She complains of the most violent colicky pains in the hypogastric region at the menstrual periods. The pains can only be controlled by hypodermic injections of morphia. On physical examination the abdomen was found greatly distended by a tumor in the hypogastric and umbilical regions. The vagina was found to be completely occluded at the *ostium externum*. Rectal examination disclosed a tumor supposed to be the upper portion of the vagina and uterus distended by retained menstrual fluid. The anterior portion of the tumor was soft, and was believed to be the distended upper portion of the vagina, while the firmer posterior portion was supposed to be the distended uterus.

"On the following day the patient was etherized, a sound introduced into the bladder and a finger into the rectum as guides. A vertical incision was made at the bottom of the labial fold and rapid dilation practiced with the finger and a sound until a septum was reached, which was at first believed to mark the distended upper portion of the vagina, but afterwards found to be the cervical portion of the uterus. This was punctured by sharp scissors and the imprisoned blood and secretions allowed to escape. The aperture thus made was further dilated with Peaslee's hard rubber dilators, and by separating the handles of the uterine dressing forceps previously inserted. The uterine contents were brownish-black and viscid, having the color and consistency of molasses. After the contents had been evacuated the uterus was washed out with a one-half per cent. solution of carbolic acid. A Sims' hard rubber vaginal dilator was then introduced into the vagina and the patient put to bed.

"The next day there was very slight constitutional disturbance, which soon disappeared under the use of the carbolic acid douches repeated twice a day.

"On the 17th day after the operation the patient left the hospital, well. She was wearing the vaginal dilator, which she was directed to continue using constantly and to pay attention to the cleanliness of the parts."

This was three months ago, and to-day she has returned to us complaining of symptoms similar to those she suffered from previous to her former treatment. On examination I find that the abdominal enlargement has returned, though to a much less degree than before. The vagina is pervious to the finger and feels like the lining membrane of an abscess. No hard cicatricial tissue can be detected. I can pass my finger up to the cervix, which has again become occluded, and above it is the enlarged uterus. The patient being etherized, I push a grooved director through the occluded os, which at once gives exit to a quantity of dark fluid. In order to prevent recontraction of the orifice, I introduce successively larger sizes of Peaslee's hard rubber dilators until my finger passes readily into the uterine cavity. With a one-half per cent. solution of carbolic acid the uterus is now well washed out, and an intra-uterine stem inserted. The vaginal dilator, or plug, will be again introduced and kept in place by a T bandage.

In this case the obliteration of the genital canal was undoubtedly due to injuries received during labor, three years before. Either from a prolonged labor or the injudicious use of instruments in the delivery of the child, sloughing of the vagina resulted, and no care being taken, the surfaces denuded of mucous membrane adhered to each other, producing the occlusion throughout the entire extent of the vagina. On the present occasion the atresia was confined to the os uteri, the vagina having remained pervious. The monthly discharge of blood, when the menses were re-established, was retained in the uterus, and accumulating, produced the violent, colicky pains of which the patient complained at her menstrual periods.

Women sometimes marry without being aware that such an obstacle to the consummation of the sexual act as an undeveloped vagina, exists. These cases will apply to you sometimes to perform an operation for the removal of the obstacle, by constructing a vagina. If a thorough examination from the rectum and bladder fails to demonstrate the existence of the uterus, and if in addition the symptoms of menstrual retention are absent, it is questionable whether such an operation is proper. I would advise you to undertake the operation only in cases in which life is threatened or rendered miserable by the severity of the suffering.

Palliative measures in atresia have heretofore been limited to the relief of pain at the menstrual period, and no one, so far as I am aware, has proposed anything short of an operation for the complete relief of the terrible suffering. The success which I have had, however, in a case where an operation was absolutely refused, prompts me to urge the trial, in suitable cases, of the bromide of potassium. The case I refer to is one of congenital atresia of the vagina. The patient is a young lady of 20 years of age. Menstrual molimina first showed themselves at the age of 15. The abdomen began to enlarge, and she suffered intensely at every menstrual period, requiring large doses of morphia to control the pain. She has been in my charge for three years, and now requires no morphia or other anodynes. About a week before the beginning of each menstrual period she begins taking half-drachm doses of potassium bromide three times a day. This has the effect of much reducing the hyperemia of the reproductive organs and so limiting the amount of blood poured out. In the interval between two periods sufficient of the fluid portion of the blood is absorbed to keep the tumor from growing larger. In fact, if any change is noticeable, it is that the tumor is growing smaller rather than increasing. Furthermore, she suffers very little, if any more discomfort at her menstrual periods than thousands of other women who regard themselves in perfect health. A decided objection to an operation, especially in an unmarried subject, is the great difficulty of keeping the newly formed vagina pervious. The canal having no mucous lining the opposed surfaces have a strong tendency to reunite, which necessitates the constant wearing of the vaginal plug. This becomes, at length, very irksome and leads in the end to neglect of this precaution, resulting almost certainly in the closure of the canal. It might be worth your while to try the palliative treatment in cases where an operation seems either unnecessary, exceptionally difficult, or fraught with danger to life.

[The further history of the case which furnished the subject of the foregoing lecture is as follows:

The operation set up a severe attack of pelvic cellulitis, which in a few days eventuated in an abscess of the left broad ligament. An aspirator needle was introduced and somewhat less than a pint of laudable looking pus withdrawn. Three days later there were symptoms of another abscess, but its location could not be discovered. This last abscess burst into the peritoneal cavity, causing rapid collapse, peritonitis and death. The *post mortem* examination made twenty-four hours after death showed fresh inflammatory adhesion of the peritoneum to the abdominal walls in the upper part of the abdomen and in the hypogastric region. In the right and left iliac regions there were firm adhesions of the peritoneum, evidently the remains of former inflammatory action. When the incision was extended below the umbilicus about a pint of yellowish fetid pus gushed forth. In the middle of the abdomen the peritoneum was of a black color. There was about a quart of serous exudation, containing flakes of lymph, in the peritoneal cavity. The liver was slightly adherent to the inner surface of the ribs, but normal in color. Spleen normal. Stomach distended with gas. Kidneys very slightly congested. Behind and on either side in the pelvis were two large abscesses. The one on the left side was the more superficial, and had been punctured several days before (see clinical history), but had refilled. The one situated deeper and on the right side had ruptured and emptied its contents into the peritoneal cavity. The broad ligaments, ovaries and Fallopian tubes had been matted together by the inflammatory exudation and partially disorganized. Bladder apparently normal.

The generative organs, with the bladder and rectum, were dissected out and split up in the median line. Between the bladder in front and the rectnm behind, was a fibrous canal terminating above in a small opening leading into the cervix uteri. The septum between this vaginal canal and the rectum was from one-half to one centimeter in thickness. There was not a vestige of what could properly be termed mucous membrane lining this canal. The uterus was normal in size, of a pinkish color on section, and showed slight evidences of endometritis —Rep.]

